# Deciphering complexity of GPCR signaling and modulation: implications and perspectives for drug discovery

**DOI:** 10.1042/CS20245182

**Published:** 2025-05-20

**Authors:** Claudio M. Costa-Neto, Lucas T. Parreiras-e-Silva

**Affiliations:** 1 Department of Radiology and Oncology, Faculty of Medicine, University of São Paulo (FMUSP), São Paulo, SP, Brazil; 2 Department of BioMolecular Sciences, Ribeirão Preto Pharmaceutical Sciences School (FCFRP), University of São Paulo (USP), Ribeirão Preto, SP, Brazil

**Keywords:** agonists, drug discovery and design, G protein-coupled receptors, pharmacology, renin-angiotensin system, signaling

## Abstract

G protein-coupled receptors (GPCRs) are central to pathophysiological processes and remain prominent targets in drug discovery. Recent advances in understanding GPCR signaling and modulation, such as biased agonism, dual agonism, and non-canonical G protein signaling, have expanded the therapeutic landscape of these receptors. These understandings have led (and are leading further) to innovative approaches that broaden GPCRs as therapeutic targets, going after better efficacy and minimizing adverse effects. However, tachyphylaxis, a rapid decrease in receptor responsiveness after repeated stimulation, presents a significant challenge in a chronic treatment context. Recent findings from our group revealed that tachyphylaxis in the angiotensin type 1 (AT1) receptor is primarily governed by the ligand’s dissociation rate (*k*_off_), i.e. high residence time, rather than by β-arrestin-mediated desensitization, as could be expected. This suggests that internalized AT1 receptors remain active when bound to ligands with high residence time, favoring sustained signaling from endosomes. Importantly, the concept of high residence time sheds new light on intracellular signaling phenomena and underscores the therapeutic value of modulating intracellular receptor activity, including the development of novel cell-permeant antagonists. This review discusses critical pharmacological parameters for drug discovery focused on agonists, including (i) activation of preferential signaling pathways (biased agonism), (ii) internalization/recycling rates, (iii) tachyphylaxis/desensitization, (iv) allosteric modulators, and (v) intracellular receptor signaling and its blockade, emphasizing the need for strategies that extend beyond conventional GPCRs’ functional assays. Additionally, this review highlights how advancements in high-resolution imaging, bioluminescence resonance energy transfer-based biosensors, and computational modeling are crucial for elucidating complex GPCRs’ behaviors, particularly in understanding mechanisms like tachyphylaxis and its interplay with compartment-specific signaling. These approaches not only pave the way for therapies that strategically leverage or mitigate tachyphylaxis to sustain receptor responsiveness, but could enable the design of drugs targeting intracellular pathways as a strategy to enhance efficacy and minimize adverse effects. These insights underscore the importance of integrating diverse pharmacological strategies to refine GPCR-targeted therapies and address unmet medical needs, particularly in chronic conditions where sustained receptor activity is critical.

## GPCRs activation and signaling, a general overview

G protein-coupled receptors (GPCRs) represent the largest family of membrane receptors and play a pivotal role in regulating cellular responses to external stimuli across a wide range of physiological processes, including neurotransmission, cardiovascular function, metabolism, immune responses, and many others [1,2]. While most GPCRs are activated upon ligand binding, some instead rely on ligand-independent mechanisms, such as light-induced isomerization of retinal in rhodopsins [3,4], or proteolytic cleavage in protease-activated receptors (PARs) to unveal a new N-terminal that acts as an intramolecular ligand [5,6]. Also, some receptors have been described to be activated by biophysical processes from the microenvironment, such as shear stress [7,8] and pH [9]. Moreover, several GPCRs present constitutive activity in the absence of a ligand, influencing baseline cellular signaling and playing a crucial role in both normal physiology and disease [10,11]. Understanding this phenomenon has paved the way for the development of inverse agonists, which could regulate excessive basal signaling ocurring in pathological conditions [12].

**Figure 1:**
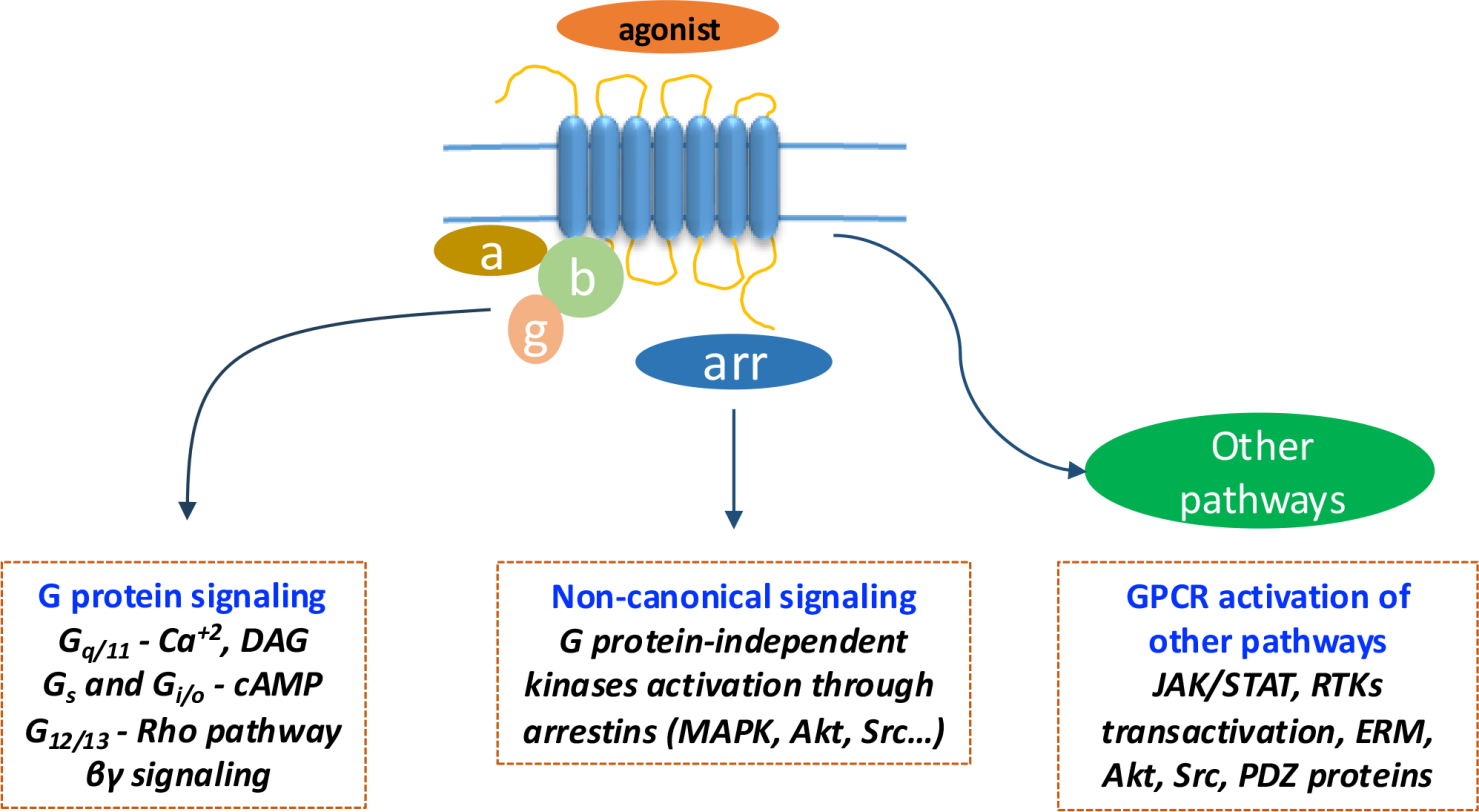
Signaling pathways mediated by GPCRs. GPCR activation can trigger different signaling routes: (1) **Classical signaling via heterotrimeric G proteins**, including Gαq/11 (generating IP₃ and DAG through PLC-β), Gαs and Gαi/o (modulating cAMP through adenylyl cyclase), and Gα12/13 (activating the Rho pathway), and βγ signaling; (2) **Non-canonical signaling**, mediated by β-arrestins, which promote the activation of kinases such as MAPK, Akt, and Src independently of G proteins; (3) **Other signaling pathways** are also described for GPCRs, including direct interaction with accessory proteins, such as PDZ-containing proteins, activation of JAK/STAT pathway and Src kinases. There are also indirect pathways, such as modulation of ERM proteins, activation of PI3K/Akt pathway and transactivation of RTKs.

Once activated, an active conformation of the receptor is stabilized, which in turn favors interaction to intracellular players and thereof activation of intracellular signaling cascades, being activation of G proteins the classical and best studied pathway [13]. Following activation, the G proteins dissociate into Gα subunits and Gβγ dimers, triggering intracellular signaling cascades [14], and accessory proteins, such as the regulators of G protein signaling (RGS) family, also influence these processes [15]. Currently, approximately, 35% of all marketed drugs target this family of receptors [16], reflecting their key role in drug discovery and to a broad spectrum of diseases. While the Gα subunits (G_s_, G_i/o_, G_q_, and G_12/13_ families) have traditionally been the primary focus of GPCRs signaling, driving the modulation of effectors such as adenylyl cyclase or phospholipase C, more recently Gβγ dimers have emerged as key regulators in their own, particularly in the modulation of ion channels and other signaling pathways. For instance, they directly interact with and modulate G protein-gated inwardly rectifying potassium (GIRK) channels [17], influencing neuronal excitability and heart rate regulation. Additionally, Gβγ subunits play a role in the regulation of voltage-gated calcium channels [18], affecting neurotransmitter release and muscle contraction. Beyond ion channels, Gβγ dimers are involved in the activation of various signaling cascades, including the MAPK pathway [19], PI3K/Akt [20], PLCβ [21], and RhoGTPases [22], thereby influencing several biological phenomena such as cell proliferation, differentiation, survival, contraction, secretion, migration, and energy homeostasis. This intricate signaling and related modulatory networks govern diverse cellular processes involved in health and disease conditions [23–25], again rendering GPCRs as highly relevant targets in drug discovery. It is also worth mentioning that GPCRs can directly activate Src family kinases [26,27] and JAK/STAT pathway [28,29] and indirectly trigger the activation of PI3K/Akt pathway [30], ezrin, radixin, and moesin (ERM) proteins [31], and the transactivation of receptor tyrosine kinases [32,33]. A schematic overview of the main signaling pathways activated by GPCRs is depicted in Figure 1.

## New mechanism of GPCRs and G protein signaling

In recent decades, significant advances have been made toward understanding the complexity of GPCRs signaling. One area that has particularly advanced is biased agonism [34–36], where certain ligands, despite acting on the same receptor, preferentially activate a/some specific downstream signaling pathways. A notable example is the biased agonist TRV130, also known as oliceridine, developed by Trevena, which targets the μ-opioid receptor and preferentially activates G protein signaling over β-arrestin recruitment [37]. By selectively engaging the G_i/o_ pathway and minimizing β-arrestin-related signaling, TRV130 is described to provide effective pain relief with reduced adverse effects such as respiratory depression and constipation, commonly associated with traditional opioids that activate both pathways [38]. Recently approved by the FDA, oliceridine (market name Olinvyk™) is the only biased agonist to have received regulatory approval to date [39,40]. This has opened new avenues for drug development, allowing the design of compounds that could potentially increase therapeutic benefits while minimizing adverse effects through preferential activation of specific pathways.

In the case of the angiotensin II receptor (AT1R), it has been proposed that a β-arrestin-biased agonist would be preferred for treating cardiovascular diseases such as heart failure and myocardial fibrosis [41]. This preference arises because β-arrestin-biased signaling at AT1R can promote cardioprotective effects independently of G protein activation, which is often associated with detrimental outcomes such as vasoconstriction, fluid retention, and hypertrophy [42]. A β-arrestin-biased agonist for the AT1R, known as TRV027 and also developed by Trevena, was evaluated in clinical trials for the treatment of acute heart failure. However, results from the Phase 2b BLAST-AHF trial did not demonstrate significant efficacy compared with placebo [43]. As a result, the clinical development of TRV027 for this indication was discontinued. This outcome highlights the challenges of translating promising preclinical results into clinical success in complex clinical indications.

β-arrestin, besides serving as a scaffold-protein and being involved in G protein-independent signaling pathways, is also involved in internalization and reduction in receptor availability at the plasma membrane. This topic will be explored in greater detail in the following section. Additionally, the discovery of non-canonical G protein signaling, where G proteins may translocate from the plasma membrane and engage in signaling pathways from intracellular locations such as endosomes [44,45], has expanded our understanding of GPCRs functional versatility, complementing the traditional signaling mechanisms that occur at the cell surface. These breakthroughs underscore the dynamic nature of GPCRs signaling and its relevance for therapeutic interventions, as it will be discussed on the topic ‘New technologies and deeper deciphering of GPCRs’ activation and modulation’.

## Tachyphylaxis in GPCRs’ signaling

Although less understood, another critical regulatory mechanism of GPCRs function is tachyphylaxis, a phenomenon characterized by a rapid decrease in receptor responsiveness after repeated stimulation [46]. Although it is not a recent discovery, tachyphylaxis remains a complex and, to some extent, undeciphered phenomenon. Tachyphylaxis is highly relevant for the development of therapeutic agonists, as it directly affects the sustainability of clinical effects [47,48]. Therefore, avoiding tachyphylaxis is essential for maintaining long-term efficacy, as repeated dosing can lead to decreased therapeutic outcomes. Understanding the molecular mechanisms underlying tachyphylaxis is key for designing drugs that minimize desensitization and for optimizing dosing strategies, such as intermittent dosing or the use of extended-release formulations.

Tachyphylaxis has significant implications for the clinical development of GPCRs-targeting drugs, particularly in chronic conditions such as asthma, hypertension, and heart failure [49]. For example, β2-adrenergic agonists, commonly used in asthma management, often exhibit reduced efficacy with repeated use due to desensitization of β2-adrenergic receptors (β2AR), which impairs long-term therapeutic effectiveness [50,51]. This poses a major challenge in maintaining sustained drug efficacy. To address this, Robert Lefkowitz’s group explored Cmpd-6, a positive allosteric modulator (PAM) of the β2AR, which enhances the effects of traditional agonists like albuterol while reducing the risk of tachyphylaxis [52]. This discovery suggests that PAMs could become a promising strategy to improve receptor responsiveness and maintain long-term efficacy. Further evidence supporting the potential of this approach to sustain receptor activity over time comes from studies on DETQ, a PAM for the dopamine D1 receptor (D1R). In human D1R knock-in mice, DETQ maintained its ability to enhance locomotor activity even after four days of repeated dosing. In contrast, direct D1R agonists, such as A-77636, showed complete tachyphylaxis by the second day of treatment [53]. This finding underscores the potential of allosteric modulators to prevent tachyphylaxis by amplifying endogenous signaling rather than directly activating the receptor, offering sustained therapeutic benefits. Similarly, LL-00066471, a PAM for α7 nicotinic acetylcholine receptors, demonstrated reduced tachyphylaxis in preclinical models, offering a more stable therapeutic profile compared to direct agonists [54]. Its ability to enhance cognitive function and sensorimotor gating without inducing tolerance supports its potential for chronic use in treating cognitive deficits associated with dementia and schizophrenia. These findings highlight the broad utility of allosteric modulators in addressing tachyphylaxis, even beyond GPCRs. Meanwhile, research on bitter taste receptors (TAS2Rs), gustatory receptors which are also expressed in airway smooth muscle [55], suggests that TAS2R agonists induce bronchodilation with minimal tachyphylaxis [56,57], offering an alternative for long-term asthma and chronic obstructive pulmonary disease (COPD) management. Together, these studies highlight the potential of targeting alternative strategies to modulate receptor function and mitigate tachyphylaxis to optimize therapeutic outcomes in the treatment of chronic diseases.

It is important to note that genetic variations in GPCRs can influence susceptibility to tachyphylaxis, highlighting pharmacogenetics also as an important aspect for drug development. Certain receptor polymorphisms may alter receptor affinity and responses to agonists, and/or affect desensitization mechanisms, leading to varied responses in different patient populations [58]. For instance, polymorphisms in the β2AR receptor gene have been linked to differential responses to asthma medications, affecting the likelihood of tachyphylaxis [59].

Understanding the molecular basis of tachyphylaxis can guide the development of ligands with improved therapeutic profiles, as seen in studies targeting the Formyl peptide receptor type 2 (FPR2), which plays a key role in inflammation, and Relaxin/insulin-like family peptide receptor 1 (RXFP1), which regulates vascular and renal function. In a recent paper by Peng et al. [60], the pharmacological profiles of two selective FPR2 agonists, BMS-986235 and ACT-389949, were compared—while BMS-986235 has shown cardioprotective effects in models of myocardial infarction, ACT-389949 was found to induce rapid and sustained receptor internalization, ultimately leading to tachyphylaxis. Using molecular docking, the authors also identified key FPR2 amino acid residues—Leu164 (TM4), Cys176 and Thr177 (ECL2), and Leu198 (TM5)—that form specific hydrophobic interactions with ACT-389949, but not with other ligands such as BMS-986235. This distinct binding mode is associated with robust β-arrestin recruitment, leading to rapid and sustained receptor internalization that results in tachyphylaxis upon repeated stimulation, therefore shedding further light onto molecular mechanisms relevant for drug design and discovery on this receptor. Another group developed an agonist for the RXFP1 with an extended half-life, with properties of increasing renal blood flow in normotensive and hypertensive conditions. Further characterization of the R2R01 compound showed that it does not lead to tachyphylaxis, an important feature supporting its development as a potential new therapy for renal and cardiovascular diseases [61], currently undergoing Phase 2 clinical trials [62]. Although the detailed molecular interactions between R2R01 and RXFP1 have not yet been elucidated, uncovering these mechanisms in future studies certainly will provide valuable insights for the rational design of next-generation RXFP1-targeting drugs.

Overcoming tachyphylaxis is a key strategy for the development of drugs aiming at minimal receptor desensitization. One promising alternative approach could be the development of biased agonists that can selectively/preferentially activate signaling pathways less prone to cause desensitization. For example, C1-S, a Gα_s_-biased β2AR agonist [63], was shown to avoid β-arrestin-mediated desensitization, which was known to induce tachyphylaxis by traditional β2AR agonists, such as albuterol [64]. By retaining G protein signaling without engaging β-arrestin, C1-S demonstrates the potential for long-term efficacy in treating conditions like asthma, offering other avenues for therapeutic intervention.

Another interesting example of a molecule that plays with differential modulation of β-arrestin to achieve an enhanced therapeutic effect is Tirzepatide (LY3298176). It has been reported as an imbalanced and dual agonist for the glucose-dependent insulinotropic polypeptide receptor (GIPR) and the glucagon-like peptide-1 receptor (GLP-1R), which means that it binds to both receptors but eventually resulting in distinct activation profiles. Tirzepatide acts as a G protein-biased agonist on the GLP-1R, reducing β-arrestin recruitment and internalization, thereby enhancing its therapeutic efficacy. On the other hand, binding of Tirzepatide to GIPR results in strong internalization, thereby preventing further activation of this receptor from the plasma membrane [65]. In May 2022, Tirzepatide (marketed as Mounjaro in the USA) was first approved to improve glycemic control in adults with type 2 diabetes mellitus (T2DM) as an adjunct to diet and exercise. It is currently in phase III trials for heart failure, obesity, and cardiovascular disorders associated with T2DM, and in phase II trials for non-alcoholic steatohepatitis (NASH). Recently, it has also been approved under the trade name Zepbound for chronic weight management [66]. Tirzepatide nicely illustrates how understanding the molecular mechanisms of GPCRs’ functionality can be of high interest for designing drugs with pharmacological features which interplay activation and internalization/desensitization, eventually aiming at improved therapeutic profiles.

Interestingly, several GPCRs exhibit minimal or no β-arrestin recruitment upon activation [67], which may reduce their susceptibility to desensitization and internalization via classical arrestin-dependent mechanisms. Over 30 GPCRs have been reported to undergo internalization through β-arrestin-independent pathways, such as caveolin- or endophilin-mediated endocytosis [68]. Examples include the CB2 cannabinoid receptor (CB2R) [69], the parathyroid hormone 2 receptor (PTH2R), the metabotropic glutamate receptor 5 (mGluR5) [70,71], AT2R [72], RXFP1, and RXFP2 [73]. These properties may offer unique pharmacological advantages for sustained therapeutic efficacy. However, further studies are needed to establish the clinical relevance of these non-canonical regulatory mechanisms.

Bitopic ligands, which simultaneously engage both orthosteric and allosteric sites on GPCRs, have demonstrated significant potential in modulating receptor activity and reducing tachyphylaxis. By stabilizing specific receptor conformations, these ligands can enhance binding affinity, improve subtype selectivity, and mitigate rapid desensitization, addressing challenges that arise in chronic treatment contexts [74]. For dopamine receptors, particularly D2R and D3R, studies have highlighted how the extracellular vestibule can be targeted to fine-tune receptor signaling and potentially decrease tachyphylaxis [75]. In this context, bitopic ligands could stabilize receptor states that promote specific intracellular signaling pathways while avoiding internalization of plasma membrane-located receptors, which is a hallmark of tachyphylaxis. This modulation is particularly illustrative in dopamine receptors due to their involvement in complex neurophysiological functions, where overactivation or dysregulation can lead to severe pathological outcomes, such as in Parkinson’s disease or schizophrenia.

Muscarinic acetylcholine receptors also provide a compelling case for bitopic ligand application. Recent findings show that these ligands can modulate receptor function by simultaneously engaging orthosteric and allosteric sites, stabilizing receptor conformations that reduce desensitization. Specifically, bitopic ligands targeting the muscarinic receptors have demonstrated the ability to enhance receptor selectivity and signaling outcomes while preventing rapid desensitization associated with chronic stimulation [76,77]. This has critical implications for the treatment of conditions such as COPD and overactive bladder, where sustained receptor activity is necessary for therapeutic efficacy. Interestingly, tiotropium—a classical M3 antagonist used in the treatment of COPD—has been shown to bind simultaneously to a second site on the receptor. This dual-site interaction underlies its long-acting properties, effectively classifying it as a clinically used bitopic ligand, and highlighting the therapeutic potential of this strategy to reduce receptor desensitization and tachyphylaxis [78]. Furthermore, the modulation of muscarinic receptors by bitopic ligands opens new possibilities for refining therapeutic strategies by reducing off-target effects and enhancing drug tolerability [79]. These findings collectively underscore the therapeutic potential of bitopic ligands in managing tachyphylaxis across different GPCRs’ families. By combining orthosteric and allosteric modulation, bitopic ligands offer an innovative approach to optimize receptor signaling, sustain therapeutic efficacy, and minimize side effects, paving the way for novel treatment strategies for chronic diseases.

## New technologies and deeper deciphering of GPCRs’ activation and modulation

Developing drugs that harness GPCR signaling and modulation requires an in-depth understanding of the molecular interactions between ligands and GPCRs. The recent elucidation of receptor structures using techniques such as crystallography, cryo-electron microscopy (cryo-EM), and molecular dynamics simulations has provided valuable insights into how different ligands can stabilize specific receptor conformations to engage distinct signaling pathways. This structural knowledge enables the design of ligands with pharmacological profiles aiming at particular therapeutic outcomes.

Many recent technological advances have increased our ability to study GPCRs function, signaling, internalization, and also tachyphylaxis. Real-time biosensors, such as those based on bioluminescence resonance energy transfer (BRET), allow researchers to monitor GPCRs activity in live cells with unprecedented precision [80]. Additionally, cryo-EM has provided high-resolution structural insights into GPCR–ligand interactions [81,82], helping to unveil the molecular basis of tachyphylaxis. For instance, a recent study identified a G protein-biased allosteric modulator of the CB1 cannabinoid receptor (CB1R), termed ago-BAM CB-05, which selectively activates G_i_ protein signaling while reducing β-arrestin recruitment, a pathway associated with receptor desensitization and adverse effects. Using cryo-EM, researchers led by Siyuan Shen and colleagues identified an extrahelical allosteric site on CB1R where ago-BAM CB-05 binds and ultimately stabilizes a G_i_-biased conformation [83]. This discovery exemplifies how side-by-side assessment using functional assays combined with structural biology can unveil novel mechanisms. Preclinical studies demonstrated that this compound provides effective analgesia in pain models without addiction or tolerance, underpinning the therapeutic potential of combining allosteric modulation and biased agonism to develop safer, more targeted GPCR-based therapies for long-term therapies. Single-molecule studies, which track the behavior of individual receptor molecules in real time, offer another powerful tool to investigate the dynamic behavior of GPCRs, revealing how conformational transitions relate to desensitization mechanisms [84,85]. These advanced techniques are changing our understanding on GPCR regulation by showing how dynamic receptor behaviors affect signaling, allowing for more detailed investigations into mechanisms like tachyphylaxis.

**Figure 2:**
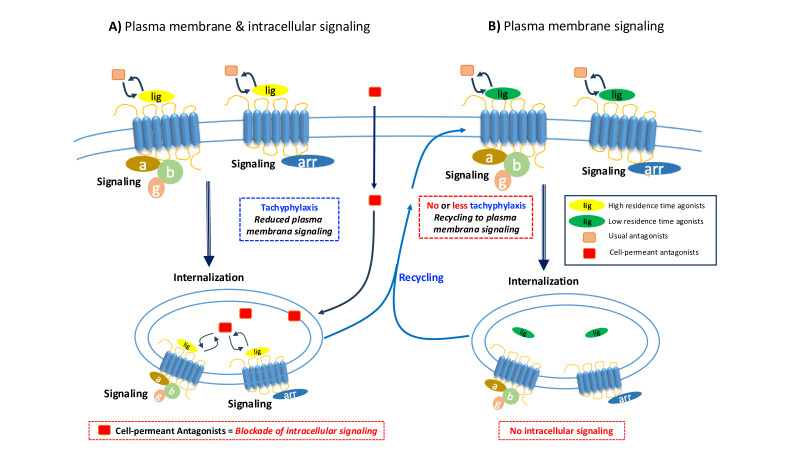
Schematic representation of certain agonists’ and receptors’ features and their implications in GPCRs’ drug discovery. (**A**) High residence time agonists trigger GPCRs signaling from plasma membrane and intracellular compartments (e.g. endosomes) due to their ability to keep receptor active conformations within these acidified vesicles after internalization. As endosomal signaling can lead to sustained GPCRs activation and possible pathological implications, an interesting approach in drug discovery could be development of cell-permeant antagonists aiming to block intracellular signaling. Such agents could also favor receptor recycling and decrease tachyphylaxis, thereby increasing receptor availability at the plasma membrane. (**B**) Conversely, low residence time agonists dissociate from the receptor during internalization, restricting receptor activation to the plasma membrane and promoting receptor recycling, thereby reducing tachyphylaxis. It is important to note that conventional antagonists block signaling at the plasma membrane, while cell-permeant antagonists inhibit both plasma membrane and intracellular signaling by also being able to reach internalized receptors. This schematic representation depicts the emerging potential of cell-permeant antagonists as a strategy for tackling intracellular signaling pathways. These insights into GPCRs’ signaling dynamics open new avenues for the discovery and development of novel therapeutics, particularly for diseases in which GPCRs’ (dys)regulation plays a critical role in their development and/or progression. GPCRs, G protein-coupled receptors.

Using BRET-based biosensors, our group has recently demonstrated that tachyphylaxis at AT1R is governed by the ligand’s dissociation rate (*k*_off_) [86], rather than by β-arrestin-mediated desensitization, as could be generally expected. Additionally, we showed that the AT1R remains active within endosomes when activated by AngII due to its low *k*_off_ (Figure 2A), while tachyphylactic analogs promoted rapid receptor recycling (Figure 2B). This finding reveals that although tachyphylactic ligands reduce the number of receptors available for activation at the plasma membrane, they simultaneously promote intracellular signaling by maintaining the internalized receptor on active conformations. Understanding the functional outcomes of this intracellular activation adds another layer of complexity on the already complex network of GPCRs signaling, but if well addressed and deciphered, it could unveil beneficial effects. In certain cases, tachyphylactic ligands might be advantageous due to their capacity to promote specific intracellular signaling pathways from distinct intracellular compartments.

Indeed, in addition to traditional signaling at the plasma membrane, GPCRs have been found to signal from intracellular compartments. For example, the receptor/β-arrestin complex can activate MAPK signaling in endosomes, leading to functional outcomes distinct from MAPK activation at the plasma membrane [33,87]. Furthermore, some receptors, such as the AT1R, can activate G proteins from endosomes [88], a phenomenon that further complicates the relationship between receptor internalization and desensitization. These insights suggest that modulating receptor levels at the plasma membrane may not always be sufficient, as internalized receptors can continue to signal from endomembranes.

## Non-canonical G protein signaling as a new target for drug discovery

The phenomenon of non-canonical G protein signaling extends beyond GPCRs coupled to G_q_ such as AT1R. In fact, the first evidence of intracellular G protein activation originated from Vilardaga’s group, particularly in studies involving G_s_-coupled receptors. Although the translocation of activated G_s_ from the plasma membrane to the cytosol has been recognized since the mid-1980s [89,90], it was Vilardaga’s pivotal work in 2009 with the parathyroid hormone receptor (PTHR) that elucidated a key mechanism: sustained cAMP production is driven by G_s_ activation from endosomes via internalized PTHR [91]. Subsequent studies have identified several other receptors capable of triggering this phenomenon, including the thyroid-stimulating hormone receptor, vasopressin V2 receptor (V2R), β2AR, among others [92–100].

In addition to Gα subunits, Gβγ dimers are gaining attention due to reports showing their roles not only at the plasma membrane but also in intracellular compartments, such as the endosomes, mitochondria, endoplasmic reticulum, and Golgi apparatus. For instance, receptor-induced translocation of Gβγ complexes to the Golgi has been shown to regulate its structure and secretion [101], highlighting the spatial and temporal specificity of G protein mechanisms. This process affects vesicular trafficking and protein secretion, maintains Golgi integrity, and ensures directional cellular responses, such as polarized secretion and migration, underscoring the compartmentalized nature of GPCR signaling. Furthermore, the identity of the Gγ subunit has been demonstrated to dictate the efficacy of Gβγ signaling, as it determines the type of prenylation, which in turn influences the duration of Gβγ dimer association with the membrane. This differential membrane retention affects the spatial and temporal dynamics of Gβγ signaling, with important functional consequences. A prolonged membrane association enhances the activation of effectors required for cytoskeletal remodeling, thereby fine-tuning the signaling pathways essential for macrophage migration and ensuring precise directional movement [102]. Considering the existence of 16 genes encoding for Gα subunits, 5 for Gβ and 12 for Gγ [103], the diversity of Gβγ complexes further underscores their ability to define the spatial and temporal bias of GPCR signaling, as the specific composition of Gβγ dimers determines their binding affinity to distinct effectors and cellular compartments. This diversity allows for precise modulation of downstream pathways, enabling compartment-specific functions such as the regulation of vesicular trafficking, cytoskeletal dynamics, and localized kinase activation. By tailoring signaling outcomes to the cellular context, Gβγ dimers contribute to the intricate control of processes such as migration, secretion, and immune responses, adding another layer of complexity to GPCRs intracellular signaling pathways [104].

GPCRs signaling within endosomes, and other intracellular compartments, often leads to sustained responses, extending pathways activation beyond the transient signals at the plasma membrane [105], a process that can significantly contribute to the development of pathological conditions. For example, signaling by β1-adrenergic receptors (β1AR) at the Golgi complex has been shown to influence cardiac function and contribute to heart disease progression [106]. Endosomal localization and activation of neurokinin-1 receptor is involved in sustained nociception, showing its potential for chronic pain treatment [107]. Also, activation of Akt by endosomal CXC chemokine receptor type 4 is associated with antiapoptotic signaling, promoting metastatic behavior of breast cancer cells [108], and ubiquitinated receptors are directed to endosomes, where they activate p38 MAPK, resulting in sustained inflammatory responses associated with chronic inflammation [109]. Considering that receptors must be activated at the plasma membrane to reach endosomes in an active conformation, these findings highlight the therapeutic potential of targeting receptor signaling within specific intracellular compartments, as it may provide opportunities to fine-tune physiological responses while minimizing off-target effects.

While these examples focus on intracellular G protein-mediated signaling, GPCRs also engage in non-canonical signaling that does not rely on either G proteins or β-arrestins. These alternative pathways are often mediated by accessory, or scaffolding proteins, which interact with specific receptor domains and shape receptor localization, stability, and function. A well-characterized example is the AT1R, which can interact with AT1R-associated protein (ATRAP). ATRAP acts as a negative regulator of AT1R signaling by promoting receptor internalization and down-regulation [110], thereby dampening Ang II-induced hypertrophic and fibrotic responses in cardiovascular tissues. In contrast, AT2R-interacting protein (ATIP) binds to the AT2R and facilitates its signaling toward cell growth inhibition and neurite outgrowth, independently of classical G protein pathways [111–113]. Beyond ATRAP and ATIP, other proteins such as spinophilin [114], PDZ domain-containing proteins [115,116], and G protein-coupled receptor kinase interactor 1 (GIT1) [117,118] have been shown to scaffold specific GPCRs, modulate their subcellular localization, recycling, and degradation, or bias their signaling outputs toward selective intracellular pathways, revealing an added layer of regulation that is both receptor- and cell type-specific. Altogether, these findings underscore the growing appreciation that GPCR signaling is profoundly shaped by intracellular context and accessory protein networks.

Our group recently demonstrated that Gs activation by internalized V2R is dependent on ligand stimulation with a low *k*_off_ [119], emphasizing the critical role of ligand–receptor kinetics in shaping intracellular signaling outcomes. We also found that β-arrestin plays a dual role in regulating endosomal V2R signaling. It not only escorts the receptor to endosomes, favoring signaling at this compartment, but also accelerates receptor inactivation due to ligand displacement in these acidified vesicles [119]. This finding supports the notion that avoiding the β-arrestin pathway not only increases receptor availability at the plasma membrane but also reduces compartmentalized signaling, altering downstream responses. Interestingly, we have shown that only a membrane-permeant antagonist could effectively block the intracellular activity of V2R, highlighting the therapeutic potential of targeting intracellular signaling specifically (Figure 2A).

The emerging understanding that GPCRs can signal from intracellular compartments has spurred interest in the development of cell-permeant ligands also capable of modulating these receptor pools. Several recent studies have highlighted the mechanistic and possible therapeutic relevance of such modulation. Clozapine, a clinically approved atypical antipsychotic, has been described to act on intracellular D2R, promoting their trafficking to the plasma membrane and thereby modulating dopaminergic signaling at both surface and internal sites [120]. Similarly, netupitant, a NK1 receptor antagonist used clinically in antiemetic therapy, displays enhanced potency due to its ability to access and block endosomal NK1 receptors, which are essential for sustained nociceptive signaling [121]. Beyond these clinical examples, recent studies have demonstrated the feasibility of developing novel membrane-permeant ligands specifically designed to reach intracellular GPCRs. One such case is a newly developed β1AR antagonist capable of blocking Golgi-localized β1AR signaling, thereby affecting a kind of receptor functionality which impermeant antagonists are unable to tackle [122]. Another good example is the intracellular 5-HT2A receptors involved in mediation of plasticity-promoting effects of psychedelics in contrast to serotonin, which is membrane-impermeant and, therefore, is unable to activate this compartmentalized signaling [123]. These findings highlight the key potential of membrane-permeant ligands: they can access both surface and internal receptor pools, enabling more complete modulation of GPCR activity. This is especially relevant in diseases driven by sustained intracellular signaling, where impermeant drugs may be insufficient to deliver a sustained response.

## Blocking internalized GPCRs as a new therapeutic approach

As discussed above, in certain pathological conditions, GPCR internalization can play a crucial role in mediating the effects of persistent stimulation, leading to dysregulated cellular responses. Cell-permeant antagonists are able to transpose the cell membrane barrier and inhibit receptor activity from within the cell, interrupting signaling at the level of the endosome or other intracellular compartments. These antagonists usually bear lipophilic or amphipathic properties, enabling them to cross the plasma membrane, and once inside the cell, they can potentially reach endosomal compartments where internalized GPCRs are localized.

By preventing the activation of G proteins or β-arrestins, and other signaling cascades, cell-permeant antagonists can disrupt signaling pathways originated from internalized receptors. Moreover, some cell-permeant antagonists may affect the fate of internalized GPCRs. Since endosomal receptor activation promotes their retention within this compartment [124], antagonists binding to receptors in the endosomes can redirect their trajectory, favoring recycling back to the plasma membrane over degradation. This process ultimately increases the receptor’s overall presence at the cell surface.

Specific goals in the development of intracellular-acting antagonists could include the following:

1. Many GPCRs, such as the β2AR or the AT1R, undergo extensive internalization and participate in endosomal signaling that can contribute to disease pathogenesis. By also targeting these receptors inside the cell, antagonists could block pathogenic intracellular (over)activation. This blockade could have important therapeutic implications in disorders where aberrant endosomal signaling contributes to disease.
2. Certain GPCRs exhibit a predominant intracellular localization, challenging the classical view of their presence at the plasma membrane [125]. For example, mGluR5 is abundantly expressed in the endoplasmic reticulum, where it interacts with IP3 receptors to modulate calcium homeostasis, influencing synaptic plasticity and contributing to neuropathic pain [126–128]. Similarly, the melatonin MT1 receptor and CB1R are localized at the outer mitochondrial membrane, where they inhibit adenylyl cyclase via G_i_ proteins. This regulation influences essential organelle functions, including energy production, oxidative stress management, and dynamics related to fusion and fission [129,130]. Cell-permeant antagonists, in theory, can traverse the plasma membrane and gain access to GPCRs located on various endomembranes. However, these compounds may also act on receptors expressed at the plasma membrane, which could dilute their specificity. To address this, targeted strategies have been developed to selectively block receptor signaling from endosomes. For instance, antagonists conjugated with polyethylene glycol and cholestanol have demonstrated the ability to accumulate within endosomal vesicles, effectively inhibiting the intracellular signaling of receptors, such as NK1R, calcitonin receptor-like receptor, and PAR2(100,107,131). Expanding this approach, it would be valuable to design antagonists specifically tailored to target GPCRs in other organelles, such as the endoplasmic reticulum or mitochondria. This could be achieved by conjugating antagonists with organelle-targeting moieties, such as triphenylphosphonium groups to direct compounds to mitochondria [132] or specific retention sequences, such as the KDEL motif, to target the endoplasmic reticulum [133,134]. Such strategies would enable precise modulation of compartment-specific signaling pathways, advancing therapeutic specificity.
3. Aberrant GPCR signaling has been implicated in various pathologies, including cancer metastasis, neurological disorders, and cardiovascular diseases. The use of cell-permeant antagonists to block internalized receptors could serve as a novel therapeutic strategy to intervene in disease processes driven by abnormal GPCR activity, potentially providing more effective treatments compared with traditional membrane-only-targeted therapies.

Nevertheless, despite promising, the development of such drugs is not trivial. Since the endosomal compartments are dynamic and varied, it may be difficult to design antagonists that preferentially target receptors in specific endosomal subtypes. Additionally, the rapid recycling of certain GPCRs could limit the time window during which antagonists are effective. Finally, cell-permeant antagonists need to exhibit favorable pharmacokinetics, such as efficient intracellular delivery and sustained action within cells.

### Correlating *k*_off_, tachyphylaxis and intracellular signaling to tap new perspectives into GPCR’s agonist therapeutics

Our data obtained with V2R not only highlight the relevance of ligand-receptor kinetics but also underscore the importance of considering a drug’s partition coefficient (logP) during the processes of drug discovery and development. While logP is a key parameter as part of the Lipinski’s ‘rule of 5’ [135], it is often overlooked and underestimated in the context of extracellular targets that become internalized. Permeable antagonists, guided by their partition coefficient, can access and act on internalized receptors, expanding their therapeutic potential beyond traditional extracellular signaling modulation, as discussed above. Integrating both kinetic aspects and logP, early in the drug development process, could become key for designing drugs that effectively modulate intracellular receptor activity. This approach offers another layer of control over receptor activity, both at the membrane and inside the cell, and presents untapped therapeutic opportunities, which in turn require approaches beyond conventional surface-based assays.

Future research should investigate how ligand–receptor interactions within endosomal compartments affect receptor desensitization and activation of different signaling pathways (and/or pathways with a different kinetics of activation). Eventually, such differences from location and kinetics may result in distinct functional and (patho)physiological outcomes. Such differences need to be carefully weighed during drug discovery and development when favoring one signaling location over another. Based on our data with AT1R [86], it appears that tachyphylaxis ultimately results in intracellular signaling, suggesting that modulating these pathways could potentially shift therapeutic responses. Targeting intracellular signaling pathways might offer a way to harness beneficial aspects of this compartmentalized signaling to maintain or enhance therapeutic efficacy. The advancement of the powerful techniques mentioned above (e.g., high-resolution imaging and BRET) and integration to computational modeling will be crucial for deciphering these processes and translation into next generations of GPCRs-targeted therapeutics covering a multi-dimensional signaling spectrum.

Therefore, exploring how tachyphylaxis and intracellular signaling are interconnected can be an intriguing avenue for the development of novel types of agonistic therapeutics (Figure 2). In this mini-review, we describe pharmacological parameters of relevance for drug discovery focused on agonists and offer perspectives on their integration. Examples of parameters discussed include (i) activation of preferential signaling pathways (biased agonism), (ii) internalization/recycling rates, (iii) tachyphylaxis/desensitization, (iv) allosteric modulators, and (v) intracellular receptor signaling and its blockade. Other aspects such as ligand half-life, controlled systemic release, and multi-target agonists were not included here but comprise as well important aspects of ongoing research and innovation in GPCR drug discovery.

**Clinical Perspectives**

- **Intracellular GPCR signaling**: While one-third of all marketed drugs target GPCRs, traditional drug discovery has focused on receptors at the plasma membrane, overlooking sustained signaling from internalized receptors that may drive pathological conditions.
- **Tachyphylaxis and efficacy loss**: Some drugs promote exacerbated receptor internalization, leading to tachyphylaxis and reduced responsiveness over time, which undermines therapeutic outcomes in chronic conditions such as asthma, heart failure, and depression.
- **Innovative drug strategies**: Emerging approaches—such as allosteric modulators, biased agonists, and cell-permeant antagonists—aim to exploit intracellular signaling and modulation mechanisms to develop more effective and durable therapies, particularly in diseases driven by GPCR dysregulation.

## Abbreviations

ATIP: AT2R-interacting protein
AT1R: angiotensin II type 1 receptor
ATRAP: AT1R-associated protein
Akt: protein kinase B
BRET: bioluminescence resonance energy transfer
CB1R: cannabinoid receptor 1
D1R: dopamine 1 receptor
ERK1/2: extracellular signal-regulated Kinase 1 and 2
ERM: ezrin, radixin, and moesin
FPR2: formyl peptide receptor 2
GIT1: G protein-coupled receptor kinase interactor 1
GLP-1R: glucagon-like peptide 1 receptor
GPCRs: G protein-coupled receptors
Gα: G-protein alpha subunit
Gβγ: G-protein beta-gamma dimer
5-HT2A: 5-hydroxytryptamine (serotonin) receptor 2A
IP3: inositol 1,4,5-trisphosphate
JAK/STAT: Janus kinase/signal transducer and activator of transcription
MAPK: mitogen-activated protein kinase
MT1: melatonin receptor 1
NK1R: neurokinin-1 receptor
PAM: positive allosteric modulator
PARs: protease-activated receptors
PI3K: phosphoinositide 3-kinase
PLCβ: phospholipase C beta
PTHR: parathyroid hormone receptor
RGS: regulators of G protein signaling
RXFP1: Relaxin/insulin-like family peptide receptor 1
TAS2Rs: taste receptor type 2
V2R: vasopressin V2 receptor
cAMP: cyclic adenosine monophosphate
cryo-EM: cryo-electron microscopy
logP: partition coefficient
mGluR5: metabotropic glutamate receptor 5
β1-AR: beta-1 adrenergic receptor
β2-AR: beta-2 adrenergic receptor
β-arrestin: beta-arrestin
